# Buffalo State Asylum for the Insane

**Published:** 1880-11

**Authors:** 


### Editorial.

### BUFFALO STATE ASYLUM FOR THE INSANE.

FROM various circumstances incident to the construction and completion of such extensive buildings, the opening of the Asylum which was expected to take place during the month of October, has been unavoidably delayed. By resolution of the Board of Trustees, however, at a meeting recently held, the tenth of November has been definitely fixed for the reception of patients. As that time it is confidently expected that the profession in Buffalo, and indeed throughout Western New York will avail themselves of the advantages offered by this institution, which we think will be superior to any other in the country, for the treatment of mental diseases. The State may well be congratulated for its wisdom in providing such facilities for this important class of diseases.

A just pride in the success and reputation of this institution may well become the profession of Buffalo. The large experience and professional erudition of the Superintendent, Dr. Andrews, has inspired confidence in its management. In our last issue, we expressed the hope that the same wise judgement would be exercised in the selection of subordinates. We trust it may not be regarded as intrusive, if the medical profession in this city urge that one of its number, of acknowledged ability and attainments, should present a claim for the position of First Assistant Physician. The extent and variety of mental diseases treated in the asylum will present a field for study and research, which should be coveted by those ambitious to devote themselves to this important specialty. Of the names that have been suggested, we most cordially endorse that of Dr. W. D. Granger, whose special fitness for the duties of this position is well known. This appointment would give Buffalo a representative who would reflect credit upon the profession in which his ability and attainments are already acknowledged, while the asylum would secure an efficient and energetic officer.

